# 2-Benzyl­imino­methyl-4-chloro­phenol

**DOI:** 10.1107/S1600536808003802

**Published:** 2008-03-20

**Authors:** Xinli Zhang, Zongxiao Li

**Affiliations:** a Department of Chemistry, Baoji University of Arts and Sciences, Baoji, Shaanxi 721007, People’s Republic of China

## Abstract

The title compound, C_14_H_12_ClNO, is a Schiff base derived from the condensation of equimolar quanti­ties of 5-chloro­salicylaldehyde and 1-benzyl­amine. The mol­ecule has a *trans* configuration with respect to the imine C=N double bond. The N atom is involved in an intra­molecular O—H⋯N hydrogen bond.

## Related literature

For related literature, see: Ali *et al.* (2002 ▶); Cukurovali *et al.* (2002 ▶); Tarafder *et al.* (2002 ▶).
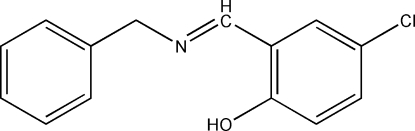


## Experimental

### 

#### Crystal data

C_14_H_12_ClNO
*M* *_r_* = 245.70
Monoclinic, 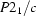

*a* = 14.3693 (18) Å
*b* = 6.0401 (8) Å
*c* = 14.777 (2) Å
β = 103.911 (2)°
*V* = 1244.9 (3) Å^3^
*Z* = 4
Mo *K*α radiation
μ = 0.29 mm^−1^
*T* = 298 (2) K
0.52 × 0.38 × 0.11 mm

#### Data collection

Bruker SMART APEX diffractometer
Absorption correction: multi-scan (*SADABS*; Sheldrick, 1996 ▶) *T*_min_ = 0.864, *T*_max_ = 0.969
5203 measured reflections
2177 independent reflections
864 reflections with *I* > 2σ(*I*)
*R*_int_ = 0.046

#### Refinement

*R*[*F* ^2^ > 2σ(*F* ^2^)] = 0.045
*wR*(*F* ^2^) = 0.116
*S* = 0.98
2177 reflections
154 parameters
H-atom parameters constrained
Δρ_max_ = 0.15 e Å^−3^
Δρ_min_ = −0.22 e Å^−3^

### 

Data collection: *SMART* (Bruker, 2000 ▶); cell refinement: *SMART*; data reduction: *SAINT* (Bruker, 2000 ▶); program(s) used to solve structure: *SHELXS97* (Sheldrick, 2008 ▶); program(s) used to refine structure: *SHELXL97* (Sheldrick, 2008 ▶); molecular graphics: *SHELXTL* (Sheldrick, 2008 ▶); software used to prepare material for publication: *SHELXTL*.

## Supplementary Material

Crystal structure: contains datablocks I, global. DOI: 10.1107/S1600536808003802/cs2068sup1.cif

Structure factors: contains datablocks I. DOI: 10.1107/S1600536808003802/cs2068Isup2.hkl

Additional supplementary materials:  crystallographic information; 3D view; checkCIF report

## Figures and Tables

**Table 1:** Hydrogen-bond geometry (Å, °)

*D*—H⋯*A*	*D*—H	H⋯*A*	*D*⋯*A*	*D*—H⋯*A*
O1—H1⋯N1	0.82	1.87	2.597 (4)	148

**Fig. 1.**
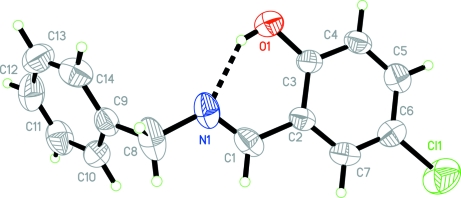
The structure of the title compound with 30% probability displacement ellipsoids. H atoms are shown as spheres of arbitrary radii. The dotted line represent a hydrogen bond.

**Fig. 2.**
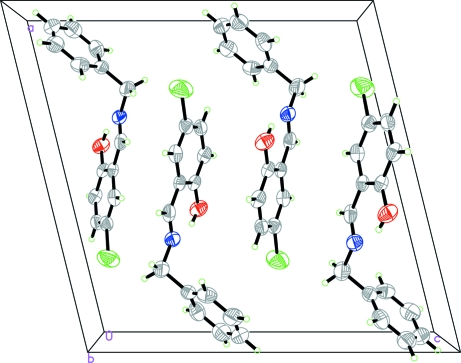
Packing of the molecules viewed along the b axis.

## supplementary crystallographic information

### Comment

Schiff base compounds have been of great interest for many years. These compounds played important role in the development of coordination chemistry related to catalysis and enzymatic reactions, magnetism and molecular architectures. These properties stimulated our interest in this field. The title compound was obtained as a new antipyrine Schiff base.

Its molecular structure and a crystal packing are illustrated in Figs.1 and 2, respectively. Atom N1 is a bridging N atom linking the two parts of the compound. The dihedral angle between the two phenyl rings is 72.91 (9) °. In the crystal structure, there exists an intramolecular O—H—N hydrogen bond involving hydroxyl atom O1 and imine atom N1 (Table 1).

### Experimental

All reagents used were of analytical grade from commercial sources and used without further purification. 5-Chlorosalicylaldehyde (0.1 mmol, 15.65 mg) and 1-benzylamine (0.1 mmol, 10.7 mg) were dissolved in methanol (10 ml). The resulting solution was stirred for 30 min, filtered and the filtrate allowed to stand at room temperature. Yellow crystals of the title compound appeared after two weeks of slow evaporation of the solvent.

### Refinement

All H atoms were placed in geometrically idealized positions and constrained to ride on their parent atoms with C—H distances in the range 0.93–0.96 Å and *U*_iso_(H) = 1.2*U*_eq_ or 1.5*U*_eq_(C/O)

### Crystal data

**Table:**

C_14_H_12_ClNO	*F*_000_ = 512
*M**_r_* = 245.70	*D*_x_ = 1.311 Mg m^−^^3^
Monoclinic, *P*2_1_/*c*	Mo *K*α radiation λ = 0.71073 Å
*a* = 14.3693 (18) Å	Cell parameters from 877 reflections
*b* = 6.0401 (8) Å	θ = 2.8–25.1º
*c* = 14.777 (2) Å	µ = 0.29 mm^−^^1^
β = 103.911 (2)º	*T* = 298 (2) K
*V* = 1244.9 (3) Å^3^	Rod, yellow
*Z* = 4	0.52 × 0.38 × 0.11 mm

### Data collection

**Table:**

Bruker SMART APEX diffractometer	2177 independent reflections
Radiation source: fine-focus sealed tube	864 reflections with *I* > 2σ(*I*)
Monochromator: graphite	*R*_int_ = 0.046
*T* = 298(2) K	θ_max_ = 25.0º
φ and ω scans	θ_min_ = 1.5º
Absorption correction: multi-scan(SADABS; Sheldrick, 1996)	*h* = −17→17
*T*_min_ = 0.864, *T*_max_ = 0.969	*k* = −7→6
5203 measured reflections	*l* = −17→10

### Refinement

**Table:**

Refinement on *F*^2^	Secondary atom site location: difference Fourier map
Least-squares matrix: full	Hydrogen site location: inferred from neighbouring sites
*R*[*F*^2^ > 2σ(*F*^2^)] = 0.045	H-atom parameters constrained
*wR*(*F*^2^) = 0.116	*w* = 1/[σ^2^(*F*_o_^2^) + (0.0248*P*)^2^ + 0.4795*P*] where *P* = (*F*_o_^2^ + 2*F*_c_^2^)/3
*S* = 0.98	(Δ/σ)_max_ < 0.001
2177 reflections	Δρ_max_ = 0.15 e Å^−^^3^
154 parameters	Δρ_min_ = −0.22 e Å^−^^3^
Primary atom site location: structure-invariant direct methods	Extinction correction: none

### Fractional atomic coordinates and isotropic or equivalent isotropic displacement parameters (Å^2^)

**Table:**

	*x*	*y*	*z*	*U*_iso_*/*U*_eq_	
Cl1	0.24166 (8)	0.1883 (2)	0.07747 (9)	0.1401 (6)	
N1	0.6895 (2)	0.0444 (5)	0.2091 (2)	0.0878 (10)	
O1	0.60143 (16)	−0.2928 (4)	0.11796 (16)	0.0884 (8)	
H1	0.6468	−0.2203	0.1478	0.133*	
C1	0.6093 (3)	0.1388 (6)	0.1982 (2)	0.0772 (11)	
H1A	0.6070	0.2803	0.2223	0.093*	
C2	0.5198 (2)	0.0337 (6)	0.1488 (2)	0.0580 (8)	
C3	0.5196 (3)	−0.1769 (6)	0.1098 (2)	0.0613 (9)	
C4	0.4338 (3)	−0.2717 (6)	0.0612 (2)	0.0738 (10)	
H4	0.4337	−0.4118	0.0350	0.089*	
C5	0.3499 (3)	−0.1585 (7)	0.0520 (2)	0.0769 (11)	
H5	0.2927	−0.2222	0.0194	0.092*	
C6	0.3489 (3)	0.0483 (7)	0.0904 (2)	0.0740 (10)	
C7	0.4331 (3)	0.1431 (6)	0.1383 (2)	0.0717 (10)	
H7	0.4320	0.2831	0.1642	0.086*	
C8	0.7753 (3)	0.1670 (8)	0.2583 (3)	0.1196 (16)	
H8A	0.8064	0.0884	0.3147	0.143*	
H8B	0.7567	0.3121	0.2759	0.143*	
C9	0.8432 (2)	0.1917 (8)	0.1974 (3)	0.0717 (10)	
C10	0.8429 (3)	0.3790 (7)	0.1443 (3)	0.0875 (12)	
H10	0.8002	0.4932	0.1469	0.105*	
C11	0.9053 (3)	0.3980 (8)	0.0877 (3)	0.1031 (15)	
H11	0.9042	0.5248	0.0517	0.124*	
C12	0.9683 (3)	0.2347 (11)	0.0834 (3)	0.1099 (16)	
H12	1.0110	0.2503	0.0454	0.132*	
C13	0.9693 (3)	0.0497 (9)	0.1342 (4)	0.1057 (15)	
H13	1.0120	−0.0640	0.1308	0.127*	
C14	0.9075 (3)	0.0294 (7)	0.1905 (3)	0.0904 (12)	
H14	0.9091	−0.0991	0.2255	0.108*	

### Atomic displacement parameters (Å^2^)

**Table:**

	*U*^11^	*U*^22^	*U*^33^	*U*^12^	*U*^13^	*U*^23^
Cl1	0.1065 (8)	0.1452 (11)	0.1546 (11)	0.0485 (8)	0.0038 (7)	0.0075 (9)
N1	0.0742 (19)	0.117 (3)	0.075 (2)	−0.030 (2)	0.0236 (18)	−0.004 (2)
O1	0.0883 (17)	0.0730 (17)	0.113 (2)	0.0039 (14)	0.0419 (15)	−0.0134 (15)
C1	0.102 (3)	0.080 (3)	0.056 (2)	−0.032 (3)	0.030 (2)	−0.014 (2)
C2	0.078 (2)	0.051 (2)	0.050 (2)	−0.007 (2)	0.0238 (18)	0.0015 (18)
C3	0.078 (2)	0.052 (2)	0.062 (2)	−0.002 (2)	0.033 (2)	0.0004 (19)
C4	0.096 (3)	0.057 (2)	0.078 (3)	−0.015 (2)	0.038 (2)	−0.015 (2)
C5	0.084 (3)	0.091 (3)	0.055 (2)	−0.016 (2)	0.016 (2)	−0.007 (2)
C6	0.082 (3)	0.077 (3)	0.063 (3)	0.014 (2)	0.017 (2)	0.011 (2)
C7	0.102 (3)	0.053 (2)	0.061 (2)	0.001 (2)	0.023 (2)	−0.0014 (19)
C8	0.092 (3)	0.186 (5)	0.086 (3)	−0.059 (3)	0.031 (3)	−0.032 (3)
C9	0.065 (2)	0.079 (3)	0.068 (3)	−0.016 (2)	0.011 (2)	−0.012 (2)
C10	0.072 (3)	0.073 (3)	0.111 (4)	0.005 (2)	0.011 (2)	−0.004 (3)
C11	0.094 (3)	0.099 (4)	0.111 (4)	−0.022 (3)	0.015 (3)	0.030 (3)
C12	0.079 (3)	0.160 (5)	0.096 (4)	−0.018 (3)	0.029 (3)	−0.002 (4)
C13	0.096 (3)	0.111 (4)	0.105 (4)	0.024 (3)	0.013 (3)	−0.021 (3)
C14	0.114 (3)	0.074 (3)	0.075 (3)	−0.004 (3)	0.007 (3)	0.003 (2)

### Geometric parameters (Å, °)

**Table:**

Cl1—C6	1.727 (3)	C7—H7	0.9300
N1—C1	1.260 (4)	C8—C9	1.485 (4)
N1—C8	1.471 (4)	C8—H8A	0.9700
O1—C3	1.349 (3)	C8—H8B	0.9700
O1—H1	0.8200	C9—C14	1.368 (5)
C1—C2	1.462 (4)	C9—C10	1.376 (5)
C1—H1A	0.9300	C10—C11	1.370 (5)
C2—C7	1.385 (4)	C10—H10	0.9300
C2—C3	1.396 (4)	C11—C12	1.351 (5)
C3—C4	1.392 (4)	C11—H11	0.9300
C4—C5	1.365 (4)	C12—C13	1.344 (5)
C4—H4	0.9300	C12—H12	0.9300
C5—C6	1.373 (4)	C13—C14	1.361 (5)
C5—H5	0.9300	C13—H13	0.9300
C6—C7	1.372 (4)	C14—H14	0.9300
			
C1—N1—C8	117.9 (4)	N1—C8—H8A	109.6
C3—O1—H1	109.5	C9—C8—H8A	109.6
N1—C1—C2	122.4 (4)	N1—C8—H8B	109.6
N1—C1—H1A	118.8	C9—C8—H8B	109.6
C2—C1—H1A	118.8	H8A—C8—H8B	108.1
C7—C2—C3	118.4 (3)	C14—C9—C10	117.2 (4)
C7—C2—C1	120.6 (3)	C14—C9—C8	121.8 (5)
C3—C2—C1	121.0 (3)	C10—C9—C8	121.0 (4)
O1—C3—C4	118.5 (3)	C11—C10—C9	120.3 (4)
O1—C3—C2	121.4 (3)	C11—C10—H10	119.9
C4—C3—C2	120.1 (3)	C9—C10—H10	119.9
C5—C4—C3	119.8 (3)	C12—C11—C10	120.8 (4)
C5—C4—H4	120.1	C12—C11—H11	119.6
C3—C4—H4	120.1	C10—C11—H11	119.6
C4—C5—C6	120.8 (3)	C13—C12—C11	119.9 (5)
C4—C5—H5	119.6	C13—C12—H12	120.1
C6—C5—H5	119.6	C11—C12—H12	120.1
C7—C6—C5	119.8 (3)	C12—C13—C14	119.7 (5)
C7—C6—Cl1	120.4 (3)	C12—C13—H13	120.2
C5—C6—Cl1	119.9 (3)	C14—C13—H13	120.2
C6—C7—C2	121.1 (3)	C13—C14—C9	122.2 (4)
C6—C7—H7	119.4	C13—C14—H14	118.9
C2—C7—H7	119.4	C9—C14—H14	118.9
N1—C8—C9	110.2 (3)		
			
C8—N1—C1—C2	178.8 (3)	C3—C2—C7—C6	−0.4 (5)
N1—C1—C2—C7	180.0 (3)	C1—C2—C7—C6	178.5 (3)
N1—C1—C2—C3	−1.2 (5)	C1—N1—C8—C9	−121.1 (4)
C7—C2—C3—O1	−179.6 (3)	N1—C8—C9—C14	−84.3 (4)
C1—C2—C3—O1	1.5 (5)	N1—C8—C9—C10	94.8 (4)
C7—C2—C3—C4	0.4 (4)	C14—C9—C10—C11	−0.1 (5)
C1—C2—C3—C4	−178.4 (3)	C8—C9—C10—C11	−179.2 (3)
O1—C3—C4—C5	179.8 (3)	C9—C10—C11—C12	−0.5 (6)
C2—C3—C4—C5	−0.2 (5)	C10—C11—C12—C13	0.9 (7)
C3—C4—C5—C6	−0.1 (5)	C11—C12—C13—C14	−0.9 (7)
C4—C5—C6—C7	0.2 (5)	C12—C13—C14—C9	0.3 (6)
C4—C5—C6—Cl1	179.8 (3)	C10—C9—C14—C13	0.2 (5)
C5—C6—C7—C2	0.1 (5)	C8—C9—C14—C13	179.3 (3)
Cl1—C6—C7—C2	−179.6 (2)		

### Hydrogen-bond geometry (Å, °)

**Table:**

*D*—H···*A*	*D*—H	H···*A*	*D*···*A*	*D*—H···*A*
O1—H1···N1	0.82	1.87	2.597 (4)	148
